# Metastatic MHC class I-negative mouse cells derived by transformation with human papillomavirus type 16

**DOI:** 10.1054/bjoc.2000.1615

**Published:** 2001-02

**Authors:** M Šmahel, E Sobotková, J Bubeník, J Šímová, R Žák, V Ludvíková, R Hájková, J Kovařík, F Jelínek, C Povýšil, J Marinov, V Vonka

**Affiliations:** Department of Experimental Virology, Institute of Hematology and Blood Transfusion, U nemocnice 1, 128 20 Prague 2, Czech Republic; Institute of Molecular Genetics, Academy of Sciences of the Czech Republic, 166 37 Prague 6, Flemingovo nám. 2, Czech Republic; Second Institute of Pathology, 1st Medical Faculty, Charles University, U nemocnice 4, 128 20 Prague 2, Czech Republic; Department of Cellular and Molecular Oncology, Masaryk Memorial Cancer Institute, Žlutý kopec 7, 656 53 Brno, Czech Republic

**Keywords:** human papillomavirus, *E6/E7* oncogenes, cell transformation, tumour-cell vaccine, DNA vaccine, H-ras

## Abstract

In the endeavour to develop a model for studying gene therapy of cancers associated with human papillomaviruses (HPVs), mouse cells were transformed with the HPV type 16 (HPV16) and activated *H-ras* oncogenes. This was done by contransfection of plasmid p16HHMo, carrying the HPV16 *E6/E7* oncogenes, and plasmid pEJ6.6, carrying the gene coding for human H-ras oncoprotein activated by G12V mutation, into secondary C57BL/6 mouse kidney cells. An oncogenic cell line, designated MK16/1/IIIABC, was derived. The epithelial origin of the cells was confirmed by their expression of cytokeratins. No MHC class I and class II molecules were detected on the surface of MK16/1/IIIABC cells. Spontaneous metastases were observed in lymphatic nodes and lungs after prolonged growth of MK16/1/IIIABC-induced subcutaneous tumours. Lethally irradiated MK16/1/IIIABC cells induced protection against challenge with 10^5^homologous cells, but not against a higher cell dose (5 × 10^5^). Plasmids p16HHMo and pEJ6.6 were also used for preventive immunization of mice. In comparison with a control group injected with pBR322, they exhibited moderate protection, in terms of prolonged survival, against MK16/1/IIIABC challenge (*P*< 0.03). These data suggest that MK16/1/IIIABC cells may serve as a model for studying immune reactions against HPV16-associated human tumours. © 2001 Cancer Research Campaign http://www.bjcancer.com


*British Journal of Cancer* (2001) **84**(3), 374–380
© 2001 Cancer Research Campaign
doi: 10.1054/ bjoc.2000.1615, available online at http://www.idealibrary.com on IDEAL®

http://www.bjcancer.com

### Metastatic MHC class I-negative mouse cells derived by transformation with human papillomavirus type 16

**M Šmahel[1], E Sobotková[1], J Bubeník[2], J Šímová[2], R Žák[1], V Ludvíková[1], R Hájková[2], J Kovařík[4], F Jelínek[1], C Povýšil[3], J Marinov[1] and V Vonka[1]**

[1]Department of Experimental Virology, Institute of Hematology and Blood Transfusion, U nemocnice 1, 128 20 Prague 2, Czech Republic; [2]Institute of Molecular Genetics, Academy of Sciences of the Czech Republic, Flemingovo nám. 2, 166 37 Prague 6, Czech Republic; [3]Second Institute of Pathology, 1st Medical Faculty, Charles University, U nemocnice 4, 128 20 Prague 2, Czech Republic; [4]Department of Cellular and Molecular Oncology, Masaryk Memorial Cancer Institute, Žlutý kopec 7, 656 53 Brno, Czech Republic

**Summary** In the endeavour to develop a model for studying gene therapy of cancers associated with human papillomaviruses (HPVs), mouse cells were transformed with the HPV type 16 (HPV16) and activated *H-ras* oncogenes. This was done by contransfection of plasmid p16HHMo, carrying the HPV16 *E6/E7* oncogenes, and plasmid pEJ6.6, carrying the gene coding for human H-ras oncoprotein activated by G12V mutation, into secondary C57BL/6 mouse kidney cells. An oncogenic cell line, designated MK16/1/IIIABC, was derived. The epithelial origin of the cells was confirmed by their expression of cytokeratins. No MHC class I and class II molecules were detected on the surface of MK16/1/IIIABC cells. Spontaneous metastases were observed in lymphatic nodes and lungs after prolonged growth of MK16/1/IIIABC-induced subcutaneous tumours. Lethally irradiated MK16/1/IIIABC cells induced protection against challenge with $10^5$ homologous cells, but not against a higher cell dose ($5 \times 10^5$). Plasmids p16HHMo and pEJ6.6 were also used for preventive immunization of mice. In comparison with a control group injected with pBR322, they exhibited moderate protection, in terms of prolonged survival, against MK16/1/IIIABC challenge ($P < 0.03$). These data suggest that MK16/1/IIIABC cells may serve as a model for studying immune reactions against HPV16-associated human tumours. © 2001 Cancer Research Campaign http://www.bjcancer.com

**Keywords**: human papillomavirus; *E6/E7* oncogenes; cell transformation; tumour-cell vaccine; DNA vaccine; H-ras

Human papillomaviruses (HPVs) are small DNA viruses that comprise over 100 types. Some of them, so-called high-risk HPVs, are oncogenic. They are aetiologically linked with cervical carcinoma (CC) and probably also with some other human cancers (zur Hausen, 1996). The type most frequently associated with human cancer is HPV16. It is detected in 50–60% of CC cases (Bosch et al, 1995).

Worldwide, CC is the second most common malignancy in women, with approximately 500 000 new cases diagnosed each year (Parkin et al, 1993). The mortality rate is about 60%. Because of the strength of the CC–HPV association, both prophylactic and therapeutic vaccines against HPVs are being developed (Vonka, 1996; Jochmus et al, 1999; van Driel et al, 1999). The only HPV proteins expressed in CC, as well as in cells transformed in vitro, are the non-structural proteins E6 and E7, which are involved both in the malignant transformation of cells and in maintenance of the transformation state (Galloway and McDougall, 1996). The expression levels of E6 and E7 correlate with their oncogenic activity (Liu et al, 1995; Trujillo and Mounts, 1996). These proteins can serve as targets of immune responses (Chen et al, 1991, 1992) induced by specific therapeutic vaccines.

Although the first clinical studies designed to cure CC by immunological intervention have already been started (McNeil, 1997a, 1997b), intensive search is still going on for suitable laboratory models in which the various parameters of immunity against HPV-induced tumours could be investigated. We reported previously the isolation of oncogenic Syrian hamster cell lines after transfection of secondary kidney cells with HPV16 *E6/E7* genes and activated *H-ras* oncogene and described specific immune reactions (both humoral and cell-mediated) in animals bearing tumours elicited by these cells (Kitasato et al, 1996). In a subsequent study (Bubeník et al, 1996), protection against challenge with these cells was induced by inoculation of irradiated homologous tumour cells, and this immunity was significantly enhanced by the simultaneous administration of mouse interleukin-2 (mIL-2); we had previously shown that mIL-2 was as effective in the hamster system as in the mouse system (Sobotková et al, 1996). In other experiments we presented evidence that protection against tumour development could also be induced by the simultaneous or preceding inoculation of HPV16-transformed hamster cells expressing the herpes simplex virus *thymidine-kinase* gene if followed by treatment with ganciclovir (GCV) (Vonka et al, 1998). Finally, we were able to induce partial immunity against these cells by immunization with plasmids carrying the HPV16 *E6/E7* genes or activated *H-ras* oncogene (Smahel et al, 1999).

In an effort to develop a system free of the major disadvantages of Syrian hamsters, i.e. their 'semisyngeneity' and a lack of reliable reagents needed for analysis of the immune responses, we recently established several HPV16-transformed mouse C57BL/6 cell lines. A protocol similar to that employed in the Syrian hamster system was used. In the present report we describe some

*Received 31 May 2000*
*Revised 14 October 2000*
*Accepted 17 October 2000*

*Correspondence to:* M Šmahel of the basic properties of the transformed mouse cells and present data from immunization/challenge experiments designed to induce protection against these cells.

### MATERIAL AND METHODS

### Animals and cells

C57BL/6 mice (H-$2^b$) (Charles Rivers, Germany) were used in this study. Animals were maintained under standard conditions and UKCCCR guidelines for the care and treatment of animals in experimental neoplasia were observed. Secondary cell cultures were prepared from the kidneys of an adult animal and grown in Dulbecco's Modified Eagle's Medium (D-MEM) (Sevac, Prague) supplemented with 10% fetal calf serum, 2 mM L-glutamine, 100 U $ml^{-1}$ penicillin and 100 µg $ml^{-1}$ streptomycin. CaSki cells have been derived from HPV16-positive human cervical cancer (Baker et al, 1987). HEF cells are spontaneously immortalized cells isolated from a hamster embryo fibroblast culture (Kutinová 1975). TC-1 cells have been prepared by transformation of C57BL/6 primary mouse lung cells with HPV-16 *E6/E7* oncogenes and activated *H-ras* (Lin et al, 1996) and were kindly provided by Dr TC Wu (Johns Hopkins University, Baltimore). All cell lines were grown in EPL medium (Sevac, Prague) (Kutinová and Vonka, 1978).

### Plasmids

Plasmids p16HHMo (Vousden et al, 1988), pEJ6.6 (Shih and Weinberg, 1982) and pAG60 (Colbere-Garapin et al, 1981), carrying, respectively, the HPV16 *E6/E7* oncogenes, the human *H-ras* oncogene activated by G12V mutation, and the neomycin resistance gene, were kindly donated by Drs K Vousden (Ludwig Institute for Cancer Research, London), M Dürst, and F Rössl (both DKFZ, Heidelberg), respectively. We compared the sequences of human and mouse H-ras proteins and found no mismatch. Therefore, the G12V mutation represents the only difference between the normal mouse H-ras protein and the human H-ras oncoprotein expressed from pEJ6.6.

Plasmids to be used in immunization experiments (see below) were propagated in *Escherichia coli*, XL1-blue strain, in Terrific Broth Medium with 100 µg $ml^{-1}$ of ampicillin added. Plasmid DNA was extracted by alkaline lysis followed by CsCl gradient centrifugation. Purified DNA was dissolved in phosphate-buffered saline (PBS) and stored at –20˚C.

### Cell transformation

Plasmids pEJ6.6 and p16HHMo were cotransfected, along with pAG60, into secondary mouse kidney cells. In the transfection, the lipofection reagent DOTAP (Boehringer, Mannheim) was used, following the manufacturer's instructions. Lines of transformed cells were established as described previously (Kitasato et al, 1996).

### Analysis of nucleic acids

DNA was extracted from cell lines by the sodium dodecyl sulphate (SDS)-proteinase K-phenol-chloroform method (Blin and Stafford, 1976). Total RNA was isolated using the RNA Blue Reagent (Top-Bio, Prague) (Chomczynski, 1993). Southern blot hybridization and the reverse transcriptase-polymerase chain reaction (RT-PCR) were performed as described previously (Kitasato et al, 1996).

### Immunoblotting

Material for the detection of the H-ras oncoprotein was prepared by lysis of cells with RIPA buffer (150 mM NaCl, 1% NP-40, 0.5% sodium deoxycholate, 0.1% SDS, 50 mM Tris, pH 7.5). Secondary mouse kidney cells (MKC) were used as a negative control. Cell monolayers in 150 $cm^2$ bottles were washed with PBS and placed on ice. One millilitre of RIPA buffer precooled at +4˚C was added per bottle and the cells were incubated on ice for 30 min with occasional rocking. Lysed cells were collected by scraping and the cell debris in the lysis buffer was transferred into an Eppendorf tube and spun at 10 000 ***g*** for 10 min at +4˚C. Samples were further analysed by 10% SDS-PAGE. The proteins separated were electroblotted onto a nitrocellulose membrane and incubated, in PBS, with anti-pan-$ras^{Val\text{-}12}$ mouse antibody (Calbiochem, La Jolla, CA) diluted in 10% non-fat milk for 2 hours at room temperature or overnight at +4˚C. The blots were then washed 5 × 5 min with PBS/0.1% Tween, secondary peroxidase-labelled anti-mouse antibody was added, and the mixture was kept for 1 hour at room temperature. The blots were washed 8 × 5 min with PBS/0.1% Tween and specific antigens were detected using the ECL Plus system (Amersham, Little Chalfont, England).

### Flow cytometry

Cells were harvested with trypsin and washed twice with PBS. For detection of MHC class I molecules, cells were incubated with anti-mouse H-$2K^b$/H-$2D^b$ monoclonal antibody (clone 28-8-6, Pharmingen, San Diego, CA) or with isotype control antibody (Sigma, St. Louis, MO) at 4˚C for 30 min, washed and incubated with FITC-conjugated goat anti-mouse Ig antibody (Pharmingen) at 4˚C for 30 min. Expression of MHC class II molecules was detected with FITC-labelled anti-mouse I-$A^b$ monoclonal antibody (clone AF6-120.1, Pharmingen).

### Immunocytochemistry

MK16/1/IIIABC and TC-1 cells grown on plastic dishes were washed with PBS and fixed in a cold mixture of methanol and acetone (1:1 by volume) for 10 min. Cytokeratins were detected by standard immunostaining with antikeratin monoclonal antibodies C-11 (binds keratins 4, 5, 6, 10, 13 and 18) and C-22 (recognizes keratins 5 and 8) (Bartek et al, 1991; Bartkova et al, 1991). Antibody DC-10 (Lauerova et al, 1988), which reacts with human keratin 18 only, was used as a negative control. Peroxidase- or FITC-conjugated rabbit antisera against mouse immunoglobulins (DAKO, Glostrup, Denmark), diluted 1:50 and 1:20, respectively, were used as secondary antibodies. Where the peroxidase conjugate was employed, positive cells were stained with DAB (Sigma) and nuclei were counterstained with haematoxylin.

### Tumour induction and growth

Cells were harvested with trypsin, washed three times with PBS and injected s.c. (0.2 ml) in the back of C57BL/6 mice. Tumour development was regularly monitored. In a DNA immunization experiment (see below), survival of tumour-bearing mice was recorded.

Student's *t*-test was used for statistical analysis.

*© 2001 Cancer Research Campaign*

### Tumour metastases

Mice were inoculated with $10^5$, $10^6$ or $10^7$ MK16/1/IIIABC cells. When their tumours reached approximately 1.5 cm in diameter, the mice were humanely killed and autopsied. Their lymph nodes, lungs, liver, heart and brain were examined histologically for the presence of spontaneous metastases. The organs were fixed in 10% neutral buffered formalin and embedded in paraffin. Sections were stained with haematoxylin and eosin.

### Immunization with irradiated cells

Male mice, 6–8 weeks old, were immunized with two doses of irradiated (200 Gy) MK16/1/IIIABC cells: $10^6$ cells were injected on day 0 and $10^7$ cells on day 34. 20 days later, groups of 5 animals were challenged s.c. with $10^5$ or $5\times10^5$ MK16/1/IIIABC cells, injected at a body site different from that used in immunization. Nonimmunized mice served as controls.

### DNA immunization

Male mice, 6–8 weeks old, received three 100 μg i.m. doses of plasmid DNA in 50 μl PBS at 3-week intervals. Plasmid pBR322 served as a negative control. 10 days after the third immunization dose, groups of 8–9 animals were challenged s.c. in the back with $10^4$ MK16/1/IIIABC cells.

### Chemicals

Cyclophosphamide (Orion Corporation Farmos, Turku, Finland) was used for treating some of the mice (300 mg $kg^{-1}$ body weight) prior to their inoculation with MK16/1 or MK16/1/IA cells.

### RESULTS

### Establishment of oncogenic mouse cell lines

Secondary kidney cells derived from C57BL/6 mice were contransfected with the following mixture of plasmids: p16HHMo, carrying the HPV16 oncogenes *E6* and *E7*; pEJ6.6, carrying the activated human *H-ras* oncogene, and pAG60, carrying the neomycin resistance gene. Following selection with the neomycin analogue G418 (200 μg $ml^{-1}$), a cell line designated MK16/1 was established. These cells ($3.3\times10^6$) were inoculated s.c. into 3-week-old mice. Tumours developed in all 5 animals treated 1 day before inoculation with cyclophosphamide, but not in any of 8 cyclophosphamide-untreated mice. Several cell lines were derived from the tumours and one of them, MK16/1/IA, was inoculated ($2\times10^6$ cells) into 3–4-month-old mice treated or untreated with cyclophosphamide. In this case, tumours developed not only in the treated animals but also in 2 of 3 untreated mice. From one of these, an MK16/1/IIAB cell line was isolated. These cells were again injected into untreated mice and several cell lines were derived from different tumours. For subsequent experiments the most oncogenic cell line, MK16/1/IIIABC, was selected. In different experiments 1 $TID_{50}$ dose ranged from $10^{3.5}$ to $10^{4.5}$, depending on the age and sex of animals. The results of a representative experiment are shown in Figure 1.

The tumours induced derived from low-differentiated epitheloid cells (Figure 2A). As angiogenesis in the tumours was low, large necroses were recorded in their centres (not shown). Despite infrequent invasive growth and the relatively rarely observed

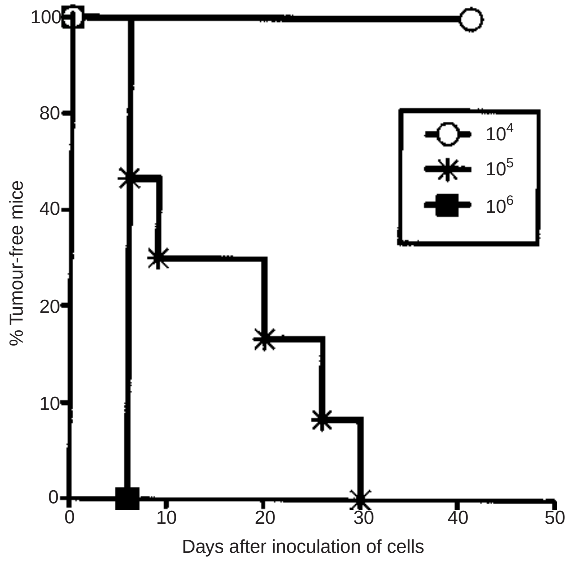

**Figure 1** Oncogenicity of MK16/1/IIIABC cells. Five-week-old female mice ($n$ = 6) were inoculated s.c. with $10^4$, $10^5$ or $10^6$ MK16/1/IIIABC cells angioinvasivity of the MK16/1/IIIABC tumours (not shown), metastases were found in draining lymphatic nodes (11–25%) and in the lungs (75–80%) (Figure 2B) after prolonged growth (7 weeks) of subcutaneous tumours induced by administration of $10^5$, $10^6$ or $10^7$ of MK16/1/IIIABC cells. This metastasizing activity apparently did not depend on the number of cells inoculated. Histological examination did not detect metastases in the liver, spleen, heart or brain.

### Nucleic-acid analysis of transformed cells

From the cell lines MK16/1, MK16/1/IA, MK16/1/IIAB and MK16/1/IIIABC, DNA and RNA were isolated. Southern blot hybridization of the DNAs, digested with the BamHI restriction enzyme, was performed using a $^{32}$P-labelled probe specific for the HPV16 *E6* gene (Figure 3A). Several bands were detected, with the band pattern being the same for all samples. This indicated stable integration of the viral DNA and a clonal origin of the cell lines.

Reverse-transcriptase PCR was used to examine unspliced and spliced forms of the E6/E7 transcripts (Figure 3B). Both unspliced E6 (PCR product, 420 bp) and spliced E6*I (238 bp) and E6*II (121 bp) forms were detected in all cell lines, with the unspliced E6 and spliced E6*I predominating. No marked differences among the different MK16/1 lines were observed.

### Detection of activated H-ras by immunoblotting

Expression of the mutated H-ras oncoprotein in MK16/1/IIIABC cells was tested by immunoblotting (Figure 3C). Antibody specific for this protein detected one protein band corresponding in size (21 kDa) to the ras product. In secondary mouse kidney cells used as a negative control, only nonmutated H-ras protein was demonstrated with anti-pan-ras monoclonal antibody (Calbiochem) (data not shown).

### Immunocytochemical detection of cytokeratins

To confirm the epithelial origin of MK16/1/IIIABC cells, expression of cytokeratins was tested. Simultaneously, TC-1 cells (Lin

© 2001 Cancer Research Campaign

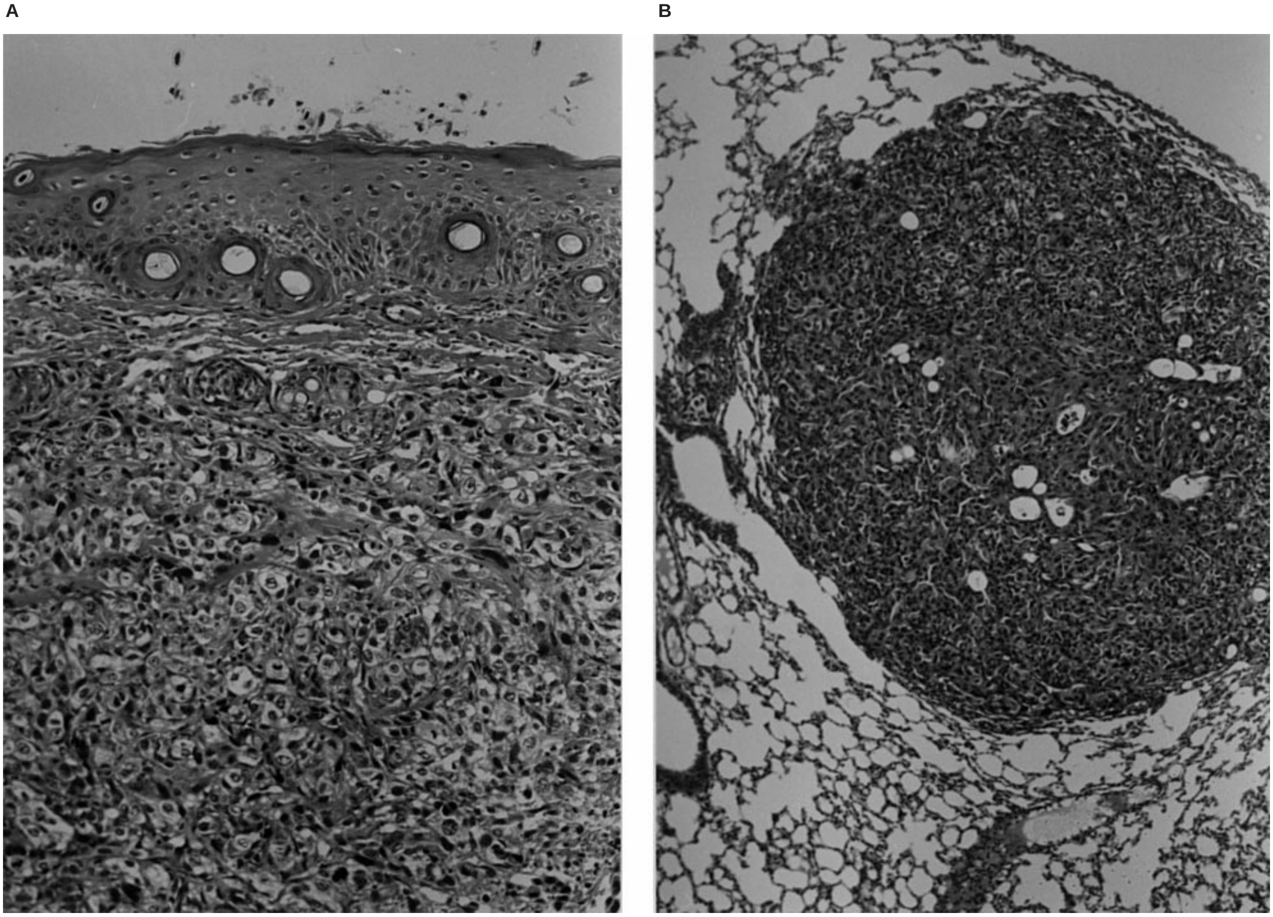

**Figure 2** Histological examination of MK16/1/IIIABC tumours. Mice were s.c. injected with $10^6$ MK16/1/IIIABC cells and humanely killed after 56 days. Sections of a paraffin-embedded primary s.c. tumour (**A**) (×150) and lung with nodular metastasis (**B**) (× 90) were stained with haematoxylin and eosin

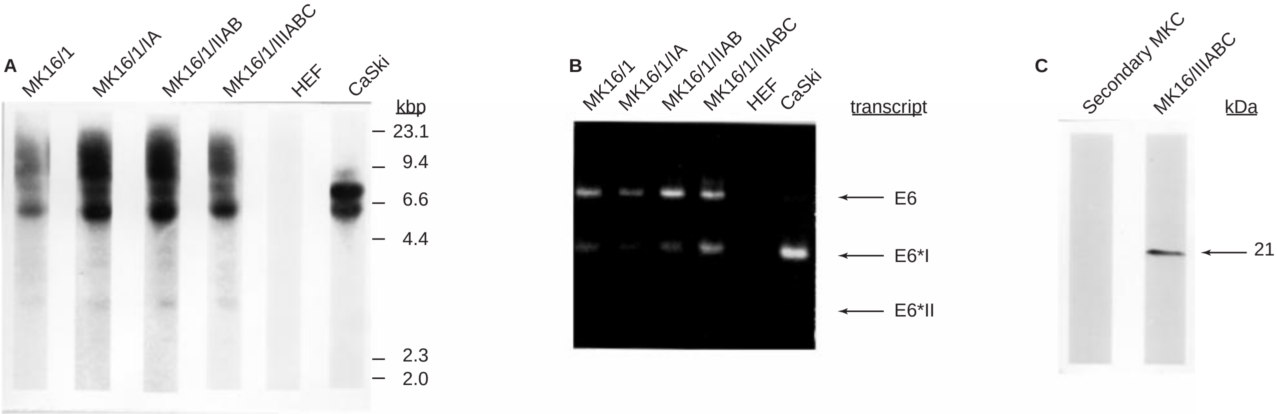

**Figure 3** (**A**) Detection of integrated HPV16 DNA by Southern hybridization. Cellular DNAs were digested with BamHI, separated electrophoretically on 1% agarose gel, and blotted onto nylon membrane. A fragment excised from the HPV16 *E6* gene (HPV16 nt 24–654) was $^{32}$P-labelled and hybridized with membrane at 65˚C for 16 hours. (**B**) Detection of E6/E7 transcripts by RT-PCR. Complementary DNA prepared from total RNA was amplified with HPV16 *E6/E7*-specific primers. The products were analysed on 3% agarose gel. Three forms of transcript were detected: E6 (420 bp), E6*1 (238 bp), and E6*II (121 bp). The smallest product, E6*II, was demonstrated in all samples but is poorly visible on the picture. (**C**) Detection of activated H-ras by immunoblotting. Whole-cell lysates were boiled in electrophoresis sample buffer, separated by 10% SDS-PAGE and transferred onto a nitrocellulose membrane. Activated H-ras was detected with mouse monoclonal anti-pan-ras$^{Val\text{-}12}$ antibody. Secondary MKC were used as a negative control et al, 1996), also transformed by HPV16 *E6/E7* and activated *H-ras* oncogenes, were analysed. MK16/1/IIIABC cells showed strong positive peroxidase staining with C-11 and C-22 antibodies, whereas the DC-10 antibody only produced a nonspecific background reaction and very weak, diffuse colouring without any apparent filamentous network. Immunofluorescence results

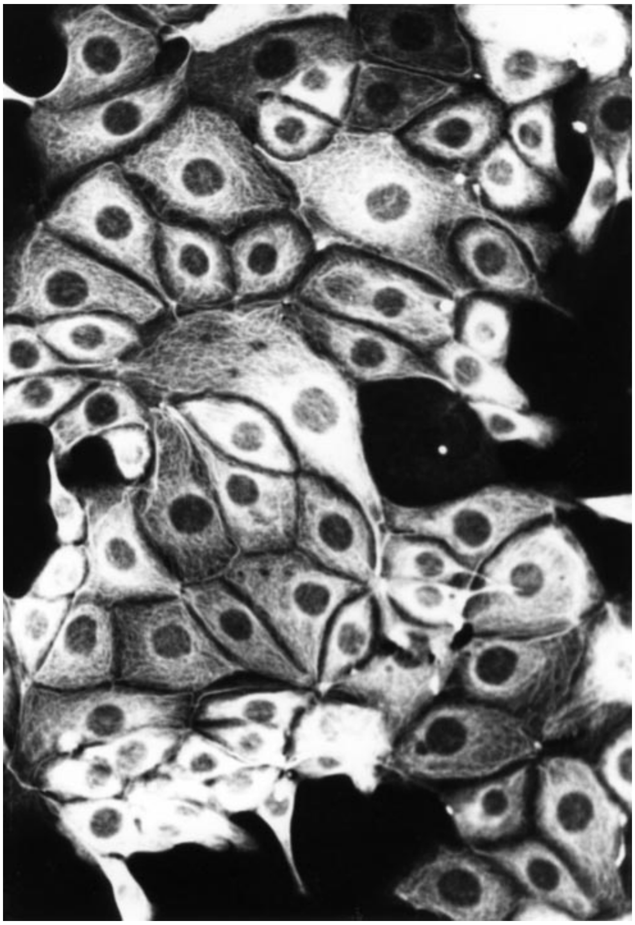

**Figure 4** Immunofluorescence visualization of keratin network in MK16/1/IIIABC cells by C-11 monoclonal antibody (× 480). Note almost homogeneous distribution of simple epithelial keratins in all cells

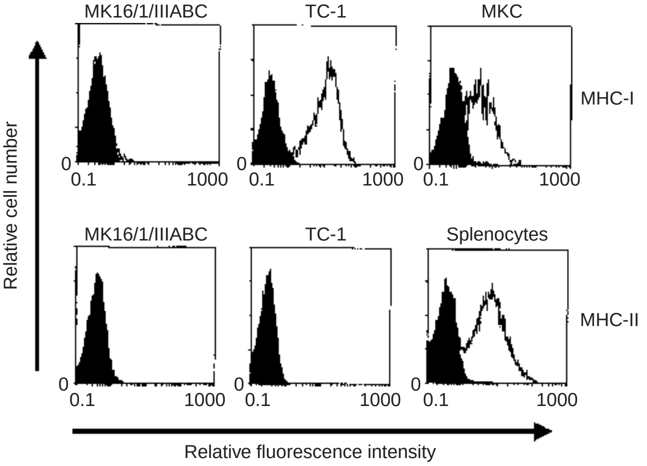

**Figure 5** Detection of MHC class I and class II molecules by flow cytometry. MK16/1/IIIABC and TC-1 cells were stained with specific monoclonal antibodies (open histograms) or with isotype control antibodies (filled histograms). Secondary MKC or splenocytes were stained as positive controls confirmed the above findings, i.e. there was strong fluorescence of keratin filaments with the C-11 (Figure 4) and C-22 (not shown) antibodies, while the DC-10 monoclonal antibody was completely negative. TC-1 cells exhibited only a diffuse background peroxidase staining with C-22 antibody but no filamentous network was seen; the C-11 and DC-10 antibodies gave negative staining. Immunofluorescence examination of TC-1 cells gave negative reactions with all of the antibodies tested.

### Testing for MHC class I and class II by flow cytometry

MHC class I and class II expression on MK16/1/IIIABC cells was examined by flow cytometry. As positive controls, secondary MKC and splenocytes were used, respectively. For comparison, TC-1 cells that had been reported to express MHC class I but not class II molecules were also stained. While secondary MKC and TC-1 cells were shown to contain MHC class I molecules, no MHC class I expression was detected on MK16/1/IIIABC cells (Figure 5). Both TC-1 and MK16/1/IIIABC cells were MHC class II negative (Figure 5).

### Induction of antitumour immunity by immunization with irradiated cells

Lethally irradiated MK16/1/IIIABC cells were administered as described in Materials and Methods to induce protection against

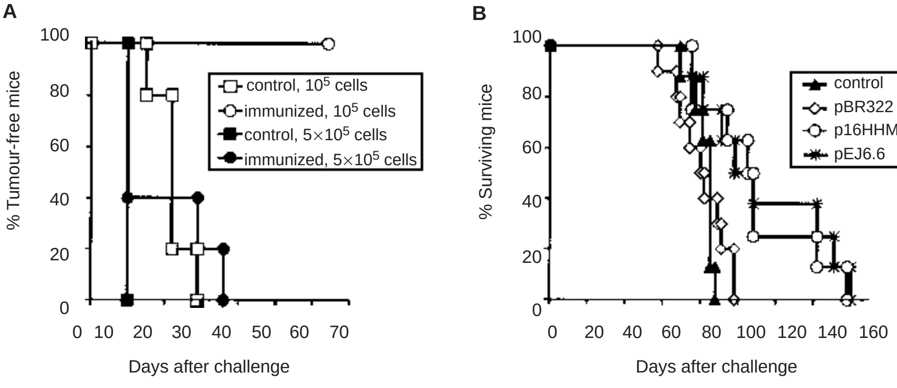

**Figure 6 (A)** Tumour formation after immunization with irradiated MK16/IIIABC cells. Mice were immunized s.c. on aday 0 with $10^6$ cells and on day 34 with $10^7$ cells. 20 days later the animals were challenged with $10^5$ or $5 \times 10^5$ homologous cells. Non-immunized mice served as a control. **(B)** Survival of mice after immunization with plasmid p16HHMo or pEJ 6.6. Mice were injected i.m. with 100 μg of plasmid DNA on days 0, 21 and 42. Plasmid pBR322 was administered as a negative control. On day 52 the animals were challenged s.c. with $10^4$ MK16/1/IIIABC cells. Tumours developed in all mice. The mean survival time of animals immunized with p16HHMo or pEJ6.6 was significantly prolonged ($P < 0.03$) in comparison with pBR322-treated animals

*© 2001 Cancer Research Campaign*

MK16/1/IIIABC challenge. This vaccination completely protected mice challenged with the lower dose ($10^5$) of tumour cells. When animals were challenged with the higher dose ($5 \times 10^5$), immunization only delayed the appearance of some tumours (Figure 6A), but the difference was not significant in comparison with control mice.

### DNA immunization against tumours

Plasmids p16HHMo and pEJ6.6, used for malignant transformation of mouse kidney cells, were each tested as a prophylactic DNA vaccine. The immunization effect in mice challenged with $10^4$ MK16/1/IIIABC cells was weak. Tumours formed in all animals, but some inhibition of tumour growth was recorded in the immunized mice and both plasmids prolonged their survival as compared with control, nonimmunized or pBR322-treated animals (Figure 6B). This difference was statistically significant ($P < 0.03$).

### DISCUSSION

An oncogenic cell line, MK16/1/IIIABC, was obtained by transformation of secondary mouse kidney cells with the HPV16 *E6/E7* and the mutated human *H-ras* oncogenes. The expression of activated H-ras in the cells was demonstrated by immunoblotting. Because of lack of reliable reagents, the E6 and E7 proteins were not detected. However, we assume they were also produced, because we showed the presence of unspliced and two spliced mRNAs that serve as transcripts for expression of both the E6 and E7 proteins. Moreover, it is reasonable to assume that without E6/E7 production oncogenic transformation of secondary mouse kidney cells would not have been achieved.

At present, TC-1 cells are increasingly utilized for the testing of anti-HPV therapeutic vaccines. Our MK16/1/IIIABC cells were obtained by transformation with the same oncogenes as TC-1 cells had been. Both cell lines originated from C57BL/6 mice; still, there are considerable differences between them. MK16/1/IIIABC were derived from secondary kidney cells, they have epitheloid morphology and strongly express a variety of cytokeratins, while TC-1 cells have been obtained from a lung cell culture, show fibroblastoid morphology and do not express any of the keratins tested in the present series of experiments. Furthermore, MK16/1/IIIABC cells are MHC class I-negative, while TC-1 cells are MHC class I-positive. Finally, subcutaneous tumours induced by MK16/1/IIIABC cells exhibited a strong metastatic potential (spontaneous lung metastases were observed in about 75% of tumour-bearing animals), while spontaneous metastases are extremely rare in animals with tumours formed after inoculation with TC-1 cells. Because of the latter two characteristics, MK16/1/IIIABC cells may serve as a highly suitable model for studying immune reactions against HPV16-associated human tumours. It should be remembered that in about 70% of CC the production of MHC class I molecules is downregulated and these patients have a worse prognosis (Connor et al, 1993; Keating et al, 1995). It has also been reported that MHC class I expression in metastases is lower than in primary tumours (Cromme et al, 1994).

We had previously shown in a hamster model that protection against tumour cells expressing HPV16 E6/E7 and activated H-ras oncoproteins could be induced by vaccination with plasmid DNA carrying either the *E6/E7* (p16HHMo) or activated *H-ras* (pEJ6.6) oncogenes (Smahel et al, 1999). Such immunization reduced tumour incidence from the 40–50% seen in the control group inoculated with pBR322 to about 10–20%. In the present model we attempted to immunize mice with the same plasmids following the same immunization scheme. However, the resulting protection was rather weak. We only recorded differences in tumour size, time of the appearance of tumours and length of survival of the animals, but not in the frequency of tumours formed. This might have been due to the properties of the challenging cells (no MHC class I and class II molecules, a low level of expression of *E6/E7* and/or *H-ras* genes) and/or a low capability of C57BL/6 mice to mount an immune response against these oncoproteins under the experimental conditions used. However, other factors might also have been involved.

As MK16/1/IIIABC cells are both MHC class I and class II negative, cytotoxic $CD^{4+}$ and $CD^{8+}$ T cells could not be responsible for the protection against the MK16/1/IIIABC challenge. However, it has been suggested that $CD4^+$ T helper cells play the central role in the immune response against tumour cells (Hung et al, 1998; Mumberg et al, 1999). This response was mediated by activated macrophages and eosinophils (Hung et al, 1998) or by indirect effects of IFN-$\gamma$ (Mumberg et al, 1999). We suppose that similar mechanisms might also be induced in our tumour system.

To summarize, we isolated a line of oncogenic, HPV16-transformed epitheloid mouse cells, free of MHC class I molecules, which metastasize spontaneously. In the present study we only managed to induce weak protection against these cells, whether by immunization with DNA coding for E6/E7 or H-ras oncoproteins, or by vaccination with irradiated homologous cells. Therefore, we are presently trying to apply approaches that might enhance immunity against tumour cells without MHC class I expression. These experiments have already been started and some results have been reported (Bubeník et al, 1999).

### ACKNOWLEDGEMENTS

We wish to thank Mrs V Navrátilová for technical assistance. This work was supported by grants Nos. NC/5900-3, NC/5526-3 and NC/45011(2)-3 from the Internal Grant Agency, Ministry of Health of the Czech Republic; by grants Nos. 312/99/0542 and 312/98/0826 from the Grant Agency of the Czech Republic, and by the Czech Terry Fox Foundation.

### REFERENCES

Baker CC, Phelps WC, Lindgren V, Braun MJ, Gonda MA and Howley PM (1987) Structural and transcriptional analysis of human papillomavirus type 16 sequences in cervical carcinoma cell lines. *J Virol* **61**: 962–971

Bartek J, Vojtesek B, Staskova Z, Bartkova J, Kerekes Z, Rejthar A and Kovarik J (1991) A series of 14 new monoclonal antibodies to keratins: characterization and value in diagnostic histopathology. *J Pathol* **164**: 215–224

Bartkova J, Bartek J, Lukas Z, Vojtesek B, Staskova Z, Bursova H, Pavlovska R, Rejthar A and Kovarik J (1991) Effects of tissue fixation conditions and protease pretreatment on immunohistochemical performance of a large series of new anti-keratin monoclonal antibodies: value in oncopathology. *Neoplasma* **38**: 439–446

Blin N and Stafford DW (1976) A general method for isolation of high molecular weight DNA from eukaryotes. *Nucleic Acids Res* **3**: 2303–2308

Bosch FX, Manos MM, Munoz N, Sherman M, Jansen AM, Peto J, Schiffman MH, Moreno V, Kurman R and Shah KV (1995) Prevalence of human papillomavirus in cervical cancer: a worldwide perspective. International biological study on cervical cancer (IBSCC) Study Group. *J Natl Cancer Inst* **87**: 796–802

Bubeník J, Simová J, Vondrys P, Vonka R, Kitasato H, Bostík P and Vonka V (1996) IL-2 as adjuvant for vaccination with cells malignantly converted by HPV 16 or 3-MC. *Int J Oncol* **8**: 477–481

© 2001 Cancer Research Campaign 

Bubeník J, Simova J, Hajkova R, Sobota V, Jandlova T, Smahel M, Sobotkova E and Vonka V (1999) Interleukin 2 gene therapy of residual disease in mice carrying tumours induced by HPV 16. *Int J Oncol* **14**: 593–597

Chen LP, Thomas EK, Hu SL, Hellstrom I and Hellstrom KE (1991) Human papillomavirus type 16 nucleoprotein E7 is a tumor rejection antigen. *Proc Natl Acad Sci USA* **88**: 110–114

Chen L, Mizuno MT, Singhal MC, Hu SL, Galloway DA, Hellstrom I and Hellstrom KE (1992) Induction of cytotoxic T lymphocytes specific for a syngeneic tumor expressing the E6 oncoprotein of human papillomavirus type 16. *J Immunol* **148**: 2617–2621

Chomczynski P (1993) A reagent for the single-step simultaneous isolation of RNA, DNA and proteins from cell and tissue samples. *Biotechniques* **15**: 532–537

Colbere-Garapin F., Horodniceanu F, Kourilsky P and Garapin AC (1981) A new dominant hybrid selective marker for higher eukaryotic cells. *J Mol Biol* **150**: 1–14

Connor ME, Davidson SE, Stern PL, Arrand JR and West CML (1993) Evaluation of multiple biological parameters in cervical carcinoma: high macrophage infiltration in HPV-associated tumours. *Int J Gynecol Cancer* **3**: 103–109

Cromme FV, van-Bommel PF, Walboomers JM, Gallee MP, Stern PL, Kenemans P, Helmerhorst TJ, Stukart MJ and Meijer CJ (1994) Differences in MHC and TAP-1 expression in cervical cancer lymph node metastases as compared with the primary tumours. *Br J Cancer* **69**: 1176–1181

Galloway DA and McDougall JK (1996) The disruption of cell cycle checkpoints by papillomavirus oncoproteins contributes to anogenital neoplasia. *Semin Cancer Biol* **7**: 309–315

Hung K, Hayashi R, Lafond WA, Lowenstein C, Pardoll D and Levitsky H (1998) The central role of CD4(+) T cells in the antitumor immune response. *J Exp Med* **188**: 2357–2368

Jochmus I, Schafer K, Faath S, Muller M and Gissmann L (1999) Chimeric virus-like particles of the human papillomavirus type 16 (HPV 16) as a prophylactic and therapeutic vaccine. *Arch Med Res* **30**: 269–274

Keating PJ, Cromme FV, Duggan KM, Snijders PJ, Walboomers JM, Hunter RD, Dyer PA and Stern PL (1995) Frequency of down-regulation of individual HLA-A and -B alleles in cervical carcinomas in relation to TAP-1 expression. *Br J Cancer* **72**: 405–411

Kitasato H, Vonka R, Bostik P, Hamsikova E, Sobotkova E, Smahel M and Vonka V (1996) Properties of Syrian hamster cells transformed by human papillomavirus type 16. *Acta Virol* **40**: 281–288

Kutinová L (1975) Growth of SV 40, adeno 7 and SV 40-adeno 7 viruses in monkey and human cells at 29 degrees and 37 degrees C. *Arch Virol* **47**: 257–268

Kutinová L and Vonka V (1978) Determination of virus-specific antigens in extracts from herpes simplex virus-infected cells by a 51Cr release inhibition test. *Inf Immun* **20**: 587–591

Lauerova L, Kovarik J, Bartek J, Rejthar A and Vojtesek B (1988) Novel monoclonal antibodies defining epitope of human cytokeratin 18 molecule. *Hybridoma* **7**: 495–504

Lin KY, Guarnieri FG, Staveley OK, Levitsky HI, August JT, Pardoll DM and Wu TC (1996) Treatment of established tumors with a novel vaccine that enhances major histocompatibility class II presentation of tumor antigen. *Cancer Res* **56**: 21–26

Liu Z, Ghai J, Ostrow RS and Faras AJ (1995) The expression levels of the human papillomavirus type 16 E7 correlate with its transforming potential. *Virology* **207**: 260–270

McNeil C (1997a) HPV vaccine treatment trials proliferate, diversify. *J Natl Cancer Inst* **89**: 280–281

McNeil C (1997b) HPV vaccines for cervical cancer move toward clinic, encounter social issues. *J Natl Cancer Inst* **89**: 1664–1666

Mumberg D, Monach PA, Wanderling S, Philip M, Toledano AY, Schreiber RD and Schreiber H (1999) CD4(+) T cells eliminate MHC class II-negative cancer cells in vivo by indirect effects of IFN-gamma. *Proc Natl Acad Sci USA* **96**: 8633–8638

Parkin DM, Pisani P and Ferlay J (1993) Estimates of the worldwide incidence of eighteen major cancers in 1985. *Int J Cancer* **54**: 594–606

Shih C and Weinberg RA (1982) Isolation of a transforming sequence from a human bladder carcinoma cell line. *Cell* **29**: 161–169

Smahel M, Sobotkova E, Vonka V, Hamsikova E, Zak R, Kitasato H and Ludvikova V (1999) DNA vaccine against oncogenic hamster cells transformed by HPV16 E6/E7 oncogenes and the activated ras oncogene. *Oncol Rep* **6**: 211–215

Sobotková E, Indrova M, Simova J, Bubenik J and Vonka V (1996) Cross-reactivity of murine and hamster IL-2 mitogenic signal. *Immunol Lett* **50**: 115–117

Trujillo JM and Mounts P (1996) Transforming activity of the E6 gene of HPV-11gt in NIH 3T3 and REF 52 cells: correlation with the level of E6 transcription. *Virology* **220**: 1–9

van Driel WJ, Kenter GG, Fleuren GJ, Melief CJ and Trimbos BJ (1999) Immunotherapeutic strategies for cervical squamous carcinoma. *Hematol Oncol Clin North Am* **13**: 259–273

Vonka V (1996) Human papillomavirus vaccines. *Folia Biol Praha* **42**: 73–78

Vonka V, Sobotkova E, Hamsikova E, Smahel M, Zak R, Kitasato H and Sainerova H (1998) Induction of anti-tumour immunity by suicide-gene-modified HPV-16-transformed hamster cells. *Int J Cancer* **77**: 470–475

Vousden KH, Doniger J, DiPaolo JA and Lowy DR (1988) The E7 open reading frame of human papillomavirus type 16 encodes a transforming gene. *Oncogene Res* **3**: 167–175

zur Hausen H (1996) Viruses in human tumors – reminiscences and perspectives. *Adv Cancer Res* **68**: 1–22

 *© 2001 Cancer Research Campaign*
